# Recurrent syncope with a type 1 Brugada electrocardiogram pattern in a patient on amitriptyline: Brugada syndrome vs. phenocopy—a case report

**DOI:** 10.1093/ehjcr/ytag710

**Published:** 2026-09-26

**Authors:** Muhammad K Asyraf, Philip Smyth, Sajjad Matiullah

**Affiliations:** Department of Cardiology, University Hospital Waterford, Dunmore Road, Waterford, X91 ER8E, Ireland; Department of Cardiology, University Hospital Waterford, Dunmore Road, Waterford, X91 ER8E, Ireland; Department of Cardiology, University Hospital Waterford, Dunmore Road, Waterford, X91 ER8E, Ireland

**Keywords:** Case report, Brugada syndrome, Brugada phenocopy, Syncope, Tricyclic antidepressant, Implantable cardioverter-defibrillator (ICD)

## Abstract

**Background:**

Brugada syndrome is an inherited arrhythmogenic channelopathy associated with an increased risk of sudden cardiac death. Diagnosis relies on the presence of a spontaneous type 1 Brugada pattern on electrocardiogram (ECG). While multiple hypotheses have been proposed to explain its underlying mechanism, it is generally thought to be secondary to defective myocardial sodium channels. For this reason, sodium channel-blocking medications can induce a type 1 Brugada pattern, which may complicate risk stratification and raise the possibility of Brugada phenocopy.

**Case summary:**

A 44-year-old man on regular amitriptyline 75 mg for anxiety and panic disorder presented with two episodes of syncope. ECG showed classical features of a type 1 Brugada pattern. Routine blood investigations, including full blood count, renal function tests, a comprehensive electrolyte panel, and high-sensitivity troponin, were normal. Transthoracic echocardiography revealed normal cardiac structure and function. Following multidisciplinary team (MDT) discussion, and guided by the 2022 European Society of Cardiology (ESC) guidelines, a shared decision was made to proceed with implantable cardioverter-defibrillator (ICD) implantation. The patient was discharged with follow-up in the arrhythmia clinic and referred for genetic testing.

**Discussion:**

This case highlighted the diagnostic and management challenges of differentiating true Brugada syndrome from drug-induced Brugada phenocopy. Although our patient had type 1 Brugada ECG pattern in the context of amitriptyline use, his presentation with syncope complicates risk stratification. MDT discussion and reference to ESC guidelines played a key role in determining further management, which in this case was ICD implantation.

Learning Points

- Sodium channel-blocking drugs, even at therapeutic doses, can induce a type 1 Brugada ECG pattern.
- Guideline-directed management, supported by MDT discussion and shared decision-making, is crucial in cases of diagnostic uncertainty.

## Introduction

Brugada syndrome (BrS) is an inherited cardiac channelopathy that increases the risk of ventricular tachyarrhythmias and sudden cardiac death in patients with structurally normal hearts. The diagnostic hallmark is the characteristic type 1 Brugada pattern on electrocardiogram (ECG), defined by a coved ST-segment elevation of ≥2 mm followed by a negative T wave in the right precordial leads V_1_ or V_2_.^1^ This pattern may be observed spontaneously or induced by sodium channel-blocking drugs or fever. BrS is diagnosed in patients with a spontaneous type 1 Brugada pattern in the absence of structural heart disease. However, diagnostic uncertainty arises when the type 1 pattern is drug-induced, as reported in 2%–4% of healthy individuals.^2^

## Summary figure

**Figure:**
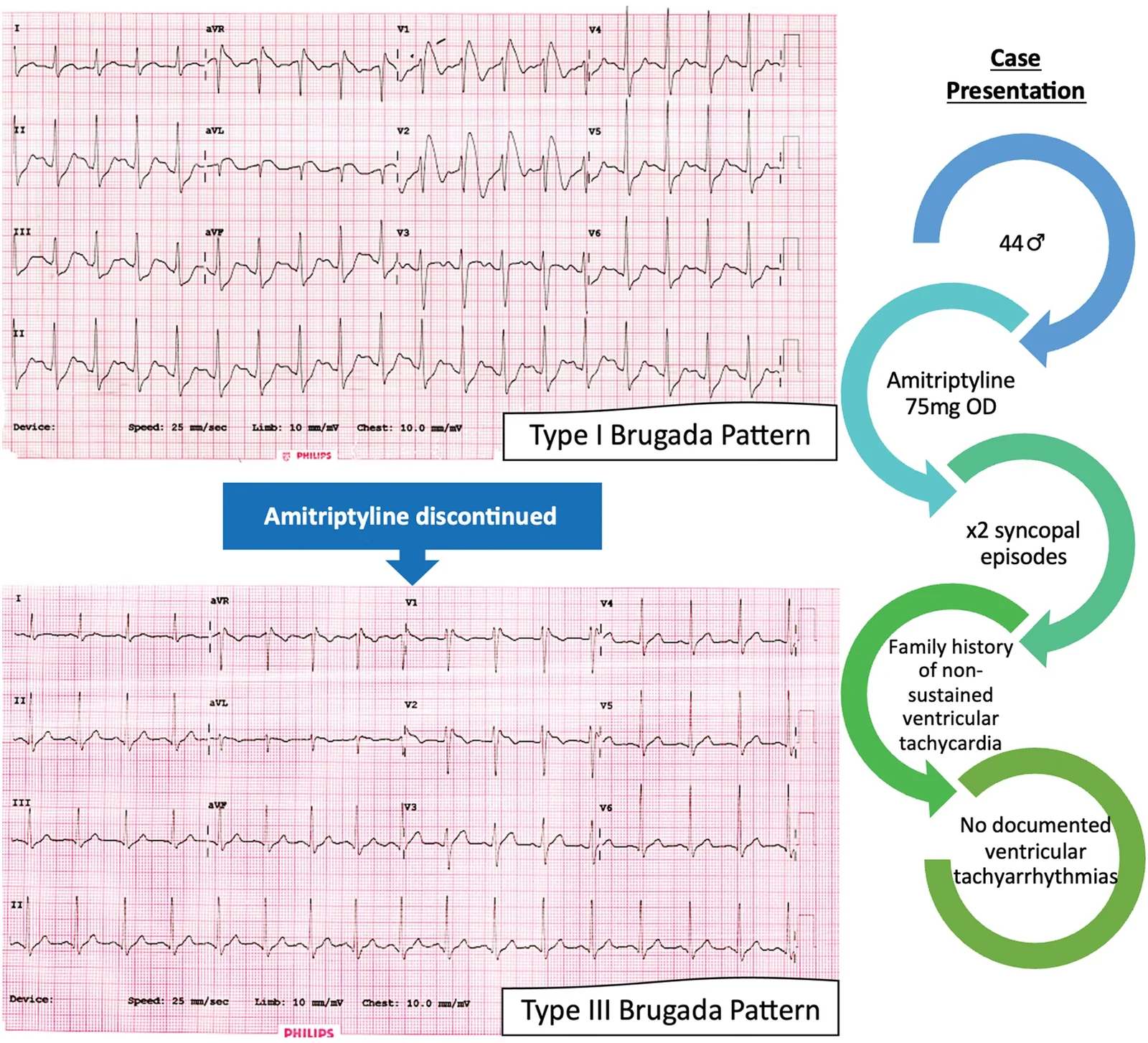


## Case presentation

A 44-year-old man presented following a witnessed syncopal episode. Collateral history revealed that he collapsed suddenly while going to the bathroom, associated with jerky arms and legs. He was unconscious for approximately 30 s, without urinary incontinence or tongue biting, but experienced some confusion lasting about 10 min before returning to baseline. Of note, he had a similar episode of witnessed syncope for 30 s while sitting on a couch watching television 6 weeks prior. He denied any prodromal symptoms during either event. His medical history is significant for anxiety and panic disorder, managed with regular amitriptyline 75 mg nocte, for the last 3 years with no recent dose adjustments. Family history is notable for a brother diagnosed with sinus node dysfunction, who underwent permanent pacemaker implantation at age 50. The same brother also had documented non-sustained ventricular tachycardia and underwent pulmonary vein isolation for atrial fibrillation, with no documented structural heart disease or Brugada pattern on ECG. There was no family history of sudden cardiac death or other cardiovascular disease.

Physical examination, including cardiovascular and full neurological assessments, was unremarkable. Serial lying-to-standing blood pressure measurements showed no significant drop. Routine blood tests, including full blood count, renal function tests, electrolytes, and troponin, were all within normal limits. Admission ECG demonstrated a classical type 1 Brugada pattern (*Figure 1*). Transthoracic echocardiography revealed normal cardiac structures. Two years ago, he underwent 24-hour Holter monitoring for intermittent mild palpitations while taking amitriptyline, which showed normal sinus rhythm throughout and no evidence of ventricular arrhythmias. He did not experience syncope at that time nor did he experience any prior syncopal events aside from the two recent episodes. No prior 12-lead ECG was available for comparison.

**Figure 1:**
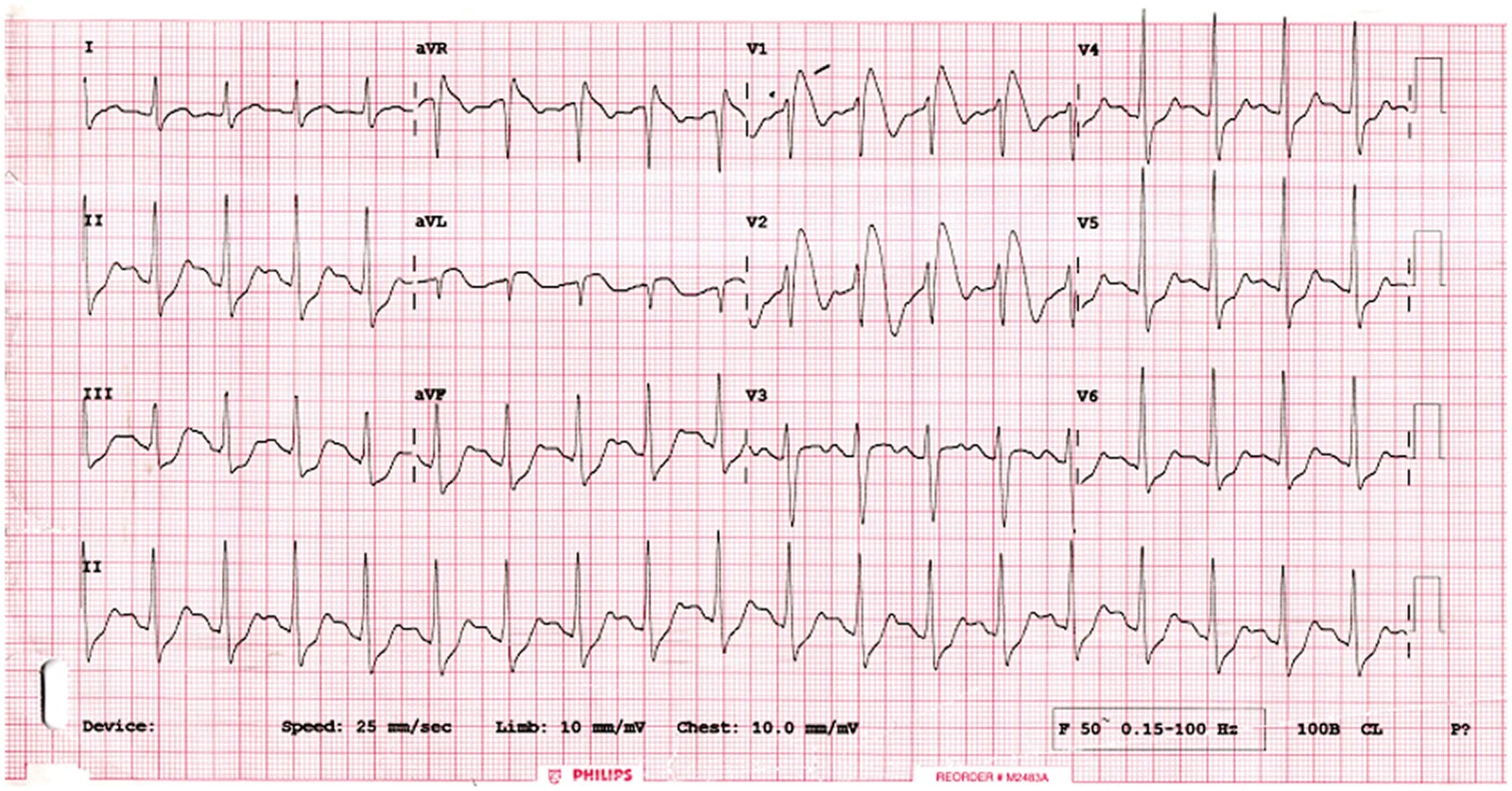
Type 1 Brugada pattern.

Amitriptyline was withheld on admission, and a repeat ECG 2 days later demonstrated a type 3 Brugada pattern (*Figure 2*). The neurology team was involved in his care, and the syncopal episodes were deemed ambiguous, with no clear cause identified on their assessment. Brain magnetic resonance imaging (MRI) performed during admission was normal, and electroencephalography was not performed. The case was discussed in a multidisciplinary team (MDT) meeting, and the patient was offered the option of implantable cardioverter-defibrillator (ICD) insertion or continuous monitoring with an implantable loop recorder (ILR). After discussion, he opted for ICD implantation. Prior to discharge, he was advised to avoid potential arrhythmia triggers associated with BrS, including cocaine, excessive alcohol intake, and certain medications (including amitriptyline, which was discontinued), and to promptly manage fever. He was referred to the arrhythmia clinic, where he remains on the waiting list for genetic testing. At 4-week follow-up, the patient has not experienced further syncopal episodes.

**Figure 2:**
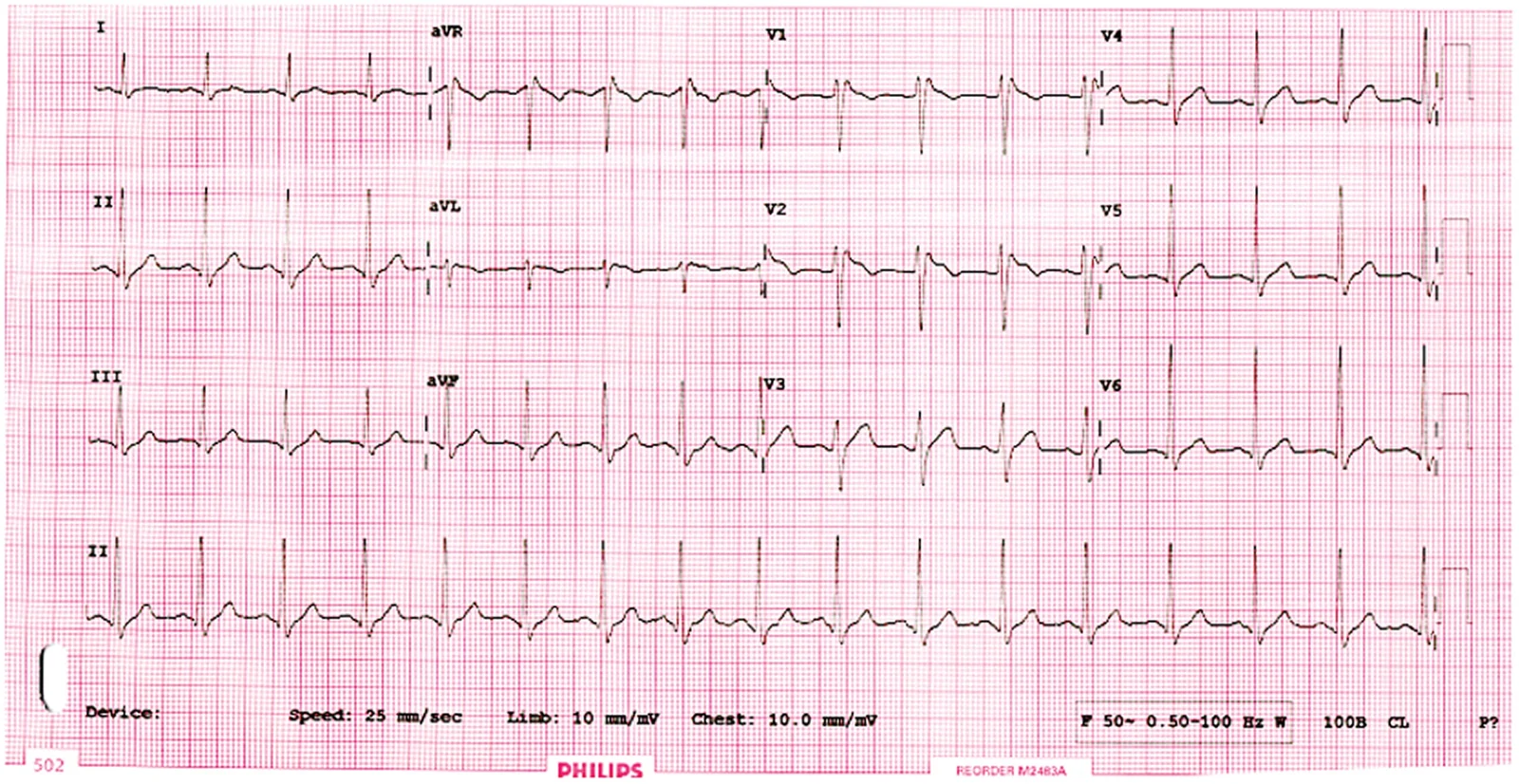
Type 3 Brugada pattern.

## Discussion

BrS is diagnosed based on a type 1 Brugada pattern on ECG in the absence of structural heart disease.^2^ As sodium channel-blocking medications such as tricyclic antidepressants can induce this pattern, distinguishing true BrS from drug-induced Brugada phenocopy can be challenging. In this case, the patient’s syncopal presentation further complicates the assessment. Differentiating arrhythmic from non-arrhythmic syncope is critical, as management strategies differ according to the 2022 European Society of Cardiology (ESC) guidelines on the management of patients with ventricular arrhythmias and the prevention of sudden cardiac death (*Figure 3*).^2^

**Figure 3:**
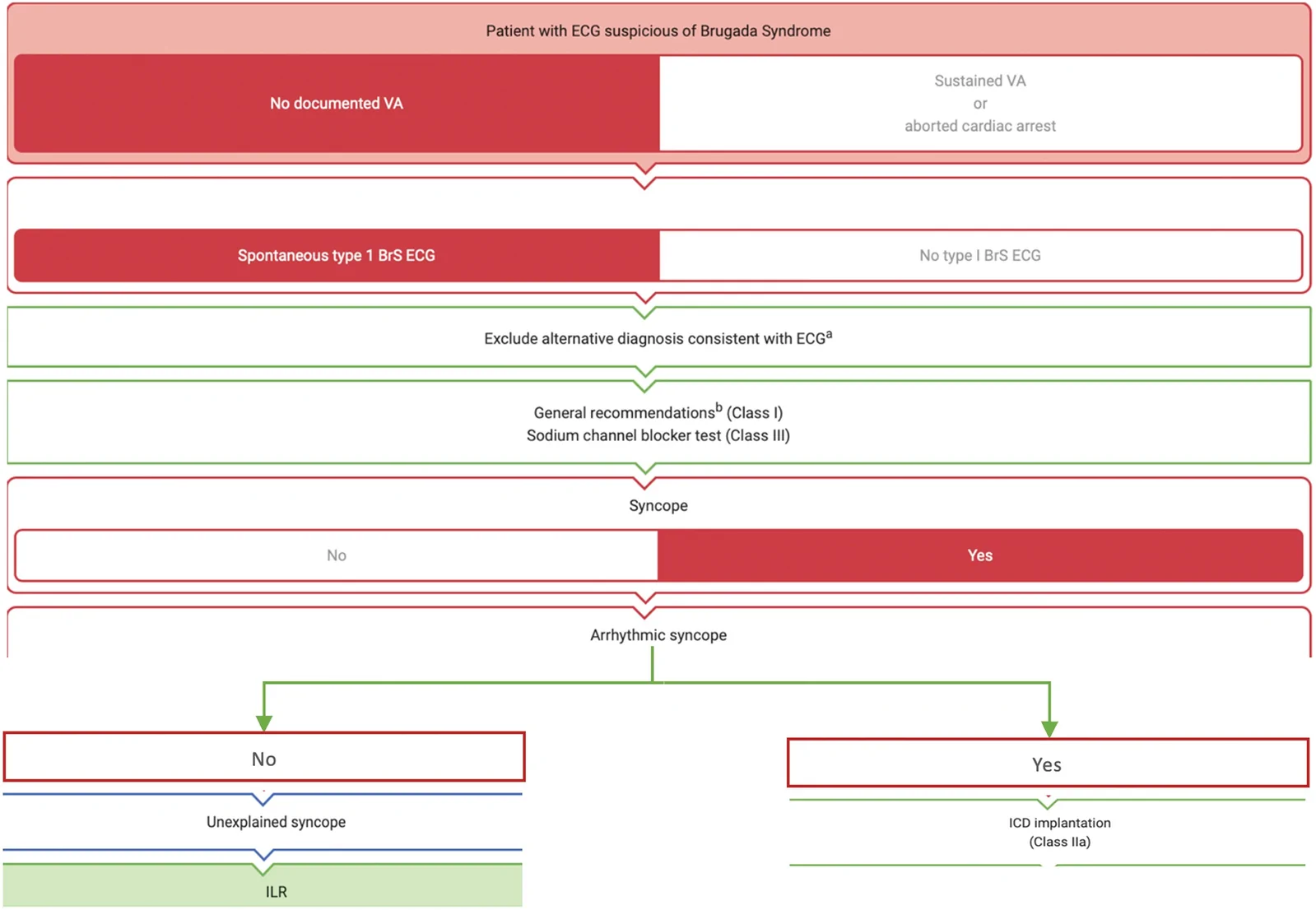
European Society of Cardiology algorithm for electrocardiogram suspicious of Brugada syndrome.

Our patient’s syncopal episodes were witnessed and associated with jerking of all four limbs. This could either represent convulsive syncope or primary seizure. Given that amitriptyline is known to lower seizure threshold,^3^ epilepsy was considered in the differential diagnosis. However, neurological assessment and brain MRI were unremarkable, and no definitive features of epilepsy were identified. Electroencephalography was not performed. The brief post-event confusion was subtle and did not clearly indicate a post-ictal state. Even after careful assessment, the cause of syncope remained ambiguous. Furthermore, there were no features in the patient history to suggest BrS, such as syncope during febrile illness or nocturnal agonal respiration. Given the diagnostic uncertainty and potential arrhythmic risk, an MDT approach involving neurology and cardiology was essential.

In our patient, the type 1 Brugada ECG pattern was observed while taking therapeutic-dose amitriptyline, suggesting a drug-induced effect. This is a unique aspect of this case, as most previously reported cases of amitriptyline-induced Brugada pattern involve overdose.^4–7^ Amitriptyline’s sodium channel-blocking properties may unmask a type 1 Brugada pattern in susceptible individuals, even at standard therapeutic doses. Importantly, the type 1 pattern resolved after discontinuation of the drug, supporting a drug-induced aetiology. However, the persistence of a type 3 Brugada pattern after drug withdrawal raises the possibility of an underlying susceptibility to BrS. This case therefore highlights the need to be aware that even therapeutic doses of tricyclic antidepressants may precipitate Brugada phenocopy or reveal underlying disease in at-risk patients.

The decision to offer ICD implantation was guided by several factors. First, the patient experienced recurrent syncope of uncertain origin, which could represent arrhythmic events, and ESC 2022 guidelines outline a Class IIa recommendation for ICD implantation in patients with suspected arrhythmic syncope and type 1 Brugada ECG pattern.^2^ Although prolonged rhythm monitoring with ILR was considered, the combination of recurrent unexplained syncope and a type 1 Brugada pattern raised sufficient concern for a potentially malignant arrhythmia to justify early intervention. In addition, the patient’s family history of ventricular arrhythmia, although non-sustained without documented Brugada pattern, raised the possibility of an underlying genetic predisposition. Genetic testing remains pending at the time of writing and was not expected to influence acute management. Taking these factors into account and following shared decision-making with the patient, ICD implantation was recommended as a precautionary strategy to mitigate the risk of sudden cardiac death in the context of suspected arrhythmic syncope and an inducible Brugada phenotype.

The main learning point from this case is that a type 1 Brugada ECG pattern alone is not always synonymous with BrS, particularly in the context of drug exposure. Further assessment with a detailed history, including the presenting complaint, drug use, and family history, alongside careful examination of ECGs, is essential for accurate diagnosis and risk stratification. In cases of diagnostic uncertainty, MDT discussion and guideline-directed management are crucial in determining appropriate management. Finally, shared decision-making with patients ensures that management aligns with their values and evidence-based recommendations, particularly in complex cases.

## Patient perspective

I was surprised to learn that the changes in my heart tracing could be linked to a serious heart problem, especially as I had been otherwise well. The uncertainty around the cause of my blackouts made the decision challenging, but after discussing the options with the cardiology team and understanding the risks and benefits, I felt that proceeding with an ICD was the safest option for me.

## Lead author biography


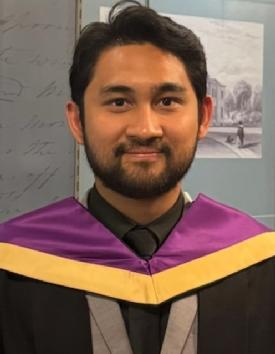
Dr Asyraf graduated from the National University of Ireland, Galway. He is currently a Cardiology Registrar at Cork University Hospital, Ireland, and an adjunct clinical lecturer with University College Cork. He has a particular interest in electrophysiology and medical education

**Consent** Appropriate informed consent was obtained from the patient for submission and publication of this case report in accordance with the Committee on Publication Ethics (COPE) guidelines.

## Data Availability

Data sharing is not applicable to this case report, as no datasets were generated or analysed. All relevant clinical information has been included.
